# Amino acid-mPEGs: Promising excipients to stabilize human growth hormone against aggregation

**DOI:** 10.22038/IJBMS.2023.67557.14804

**Published:** 2023

**Authors:** Somayeh Vakili, JamshidKhan Chamani, Zeinab Amiri-Tehranizadeh, Hossein Hosseinzadeh, Fatemeh Mosaffa, Seyed-Mola Khatami, Bahman Khameneh, Mohammad Reza Saberi

**Affiliations:** 1 Department of Medical Chemistry, School of Pharmacy, Mashhad University of Medical Sciences, Mashhad, Iran; 2 Department of Biology, Faculty of Sciences, Mashhad Branch, Islamic Azad University, Mashhad, Iran; 3 Department of Pharmacodynamics and Toxicology, School of Pharmacy, Mashhad University of Medical Sciences, Mashhad, Iran; 4 Pharmaceutical Research Center, Pharmaceutical Technology Institute, Mashhad University of Medical Sciences, Mashhad, Iran; 5 Biotechnology Research Center, Pharmaceutical Technology Institute, Mashhad University of Medical Sciences, Mashhad, Iran; 6 Department of Chemistry, Faculty of Samen Hojaj, Mashhad Branch, Technical and Vocational University (TVU), Tehran, Iran; 7 Department of Pharmaceutical Control, School of Pharmacy, Mashhad University of Medical Sciences, Mashhad, Iran

**Keywords:** Human growth hormone, Non-covalent PEGylation, Physical PEGylation, Protein formulation, Stability

## Abstract

**Objective(s):**

Today, the non-covalent PEGylation methods of protein pharmaceuticals attract more attention and possess several advantages over the covalent approach. In the present study, Amino Acid-mPEGs (aa-mPEGs) were synthesized, and the human Growth Hormone (hGH) stability profile was assessed in their presence and absence.

**Materials and Methods:**

aa-mPEGs were synthesized with different amino acids (Trp, Glu, Arg, Cys, and Leu) and molecular weights of polymers (2 and 5 KDa). The aa-mPEGs were analyzed with different methods. The physical and structural stabilities of hGH were analyzed by SEC and CD spectroscopy methods. Physical stability was assayed at different temperatures within certain intervals. Molecular dynamics (MD) simulation was used to realize the possible mode of interaction between protein and aa-mPEGs. The cell-based method was used to evaluate the cytotoxicity.

**Results:**

HNMR and FTIR spectroscopy indicated that aa-mPEGs were successfully synthesized. hGH as a control group is known to be stable at 4 ^°^C; a pronounced change in monomer degradation is observed when stored at 25 ^°^C and 37 ^°^C. hGH:Glu-mPEG 2 kDa with a molar ratio of 1:1 to the protein solution can significantly increase the physical stability. The CD spectroscopy method showed that the secondary structure of the protein was preserved during storage. aa-mPEGs did not show any cytotoxicity activities. The results of MD simulations were in line with experimental results.

**Conclusion:**

This paper showed that aa-mPEGs are potent excipients in decreasing the aggregation of hGH. Glu-mPEG exhibited the best-stabilizing properties in a harsh environment among other aa-mPEGs.

## Introduction

Nowadays, more than 130 therapeutic peptides and proteins are on the market, and their use has grown annually (1-3). Proteins have several advantages from a therapeutic perspective, including predictable intrinsic behavior and low toxicity (4). Besides these advantages, there are a few drawbacks that biotech products must overcome. Physical instabilities like denaturation or aggregation in solution lead to a biopharmaceutical formulation becoming less effective. Additionally, the structures of proteins are susceptible to environmental conditions, which means that the production, formulation, and maintenance of proteins require special attention to improve the efficacy and safety of the final formulation (5). 

One method currently employed to improve protein formulations’ physical stability involves modifying protein-stabilizing excipients, such as various sugars, sucrose, maltose, and amino acids, particularly arginine, leucine, and glutamic acid (6), polymers, and also surfactants (7). Excipients are essential components in the formulation of pharmaceutical products. They are often used to suppress protein aggregation, enhance stability and stabilize the function of unstable proteins during isolation and storage. Amino acids and polymers are frequently used in biopharmaceutical formulations to stabilize proteins and inhibit aggregation (8). Amphiphilic surfactants, can physically associate with several biotherapeutics through hydrophobic or electrostatic interactions and lead to preventing physical instabilities such as surface adsorption–induced denaturation and aggregation (9). PEG molecules bearing terminal hydrophobic moieties are used to formulate different protein drugs such as recombinant human growth hormone (hGH), granulocyte colony-stimulating factor (GCSF), and calcitonin (10). 

A novel method for stabilization against aggregation has been presented by hydrophobic interaction through noncovalent PEGylation (11). Non-covalent or physical PEGylation has shown advantages over covalent conjugation. In this case, covalent conjugation reduces the binding affinity of biotherapeutics with the target due to steric inhibition or associated structural changes (12, 13).

The theory of noncovalent PEGylation is the releasable PEGylation to the non-permit connection between PEG and protein. Thus, the linker should not remain attached to the protein’s surface. For the first time, Muller *et al*. synthesized several PEG derivatives associated with hydrophobic ligands, in which sometimes the proteins are unfolded, presented hydrophobic patches toward the solvent, and the linker of PEG to the hydrophobic group is able to interact with exposed hydrophobic protein patches (11, 14, 15). 

In this paper, hGH, as a model protein, was interacted with amino acid-mPEGs 2 and 5 kDa through non-covalent PEGylation to determine the impact of PEG or amino acids on the physicochemical properties and stability profile of hGH.

## Materials and Methods

***Materials***

This study’s buffers constituted sodium phosphate monobasic–sodium phosphate dibasic, pH 7.4, and pH 7.0. Anhydrous dichloromethane and L-tryptophan, L-Leucine, L-Arginine, L-Glutamic acid, and L-Cysteine were purchased from Fluka (Sigma-Aldrich Chemie GmbH, Buchs, Switzerland). Chloroform was supplied by Chimie-Plus (Chimie Plus Laboratoires, Denicé, France). Concentrated HCl and anhydrous Na_2_SO_4_ were obtained from Riedel de Haën (Sigma-Aldrich Laborchemikalien, Seelze, Germany). Diethyl ether, dichloromethane, anhydrous triethylamine, 2-propanol, and p-nitrophenyl chloroformate were provided by Acros (Acros Organics BVBA; Geels, Belgium). mPEG-OH 2 and 5 kDa were obtained from Iris Biotech (Iris Biotech GmbH, Marktredwitz, Germany). All solvents and compounds used were of analytical grade. Hospira, Adelaide Pty Ltd., Australia, provided Somatropin. All other chemical reagents were of analytical grade and provided by Sigma Aldrich-Fluka (Sigma, Germany).

***Methods***

*Synthesis of mPEG-p-nitrophenyl carbonate 2 and 5 kDa*

For the synthesis of *p*-nitrophenyl carbonate-mPEG (4npc-mPEG) 2 and 5 kDa, 2 mmol of M-PEG-OH, was dissolved in 20 ml of anhydrous dichloromethane, and 4 mmol of dry triethylamine (TEA) and 4 mmol of 4-nitrophenyl chloroformate (4-npc) were added under stirring while the pH was adjusted at 7.5-8.0 with Triethanolamine. 

The reaction mixture was maintained at room temperature for 24 hr. The mixture was concentrated under vacuum to about one milliliter and was dropped into 50 ml of cold diethyl ether. The precipitate was collected by filtration and re-dissolved twice in dichloromethane, precipitated from cold diethyl ether, and collected. A white powder was obtained and dried under a vacuum. The yield of M-PEG-4-npc was over 90%.

***Synthesis of amino acid-mPEGs 2 and 5 kDa***

Amino acids (20 mmol) were dissolved in 20 ml of water, and the pH was adjusted to 8.0-8.3. Then, 2 mmol of mPEG-p-nitrophenyl carbonate 2 and 5 kDa were added under stirring. The color of the reaction was yellow, and the reaction was left to proceed for 24 hr at room temperature. The solution was cooled at 0 ^°^C and reduced to pH 3 with HCl, while the reaction color became colorless. The aqueous phase was extracted three times with CHCl_3_. The chloroform was washed with water, and the organic phase was dried with anhydrous Na_2_SO_4_ and partially evaporated precipitation with diethyl ether, and the residue was crystallized by cold isopropanol. A white powder was obtained and dried under vacuum with an 85% yield.

**Figure 1:**
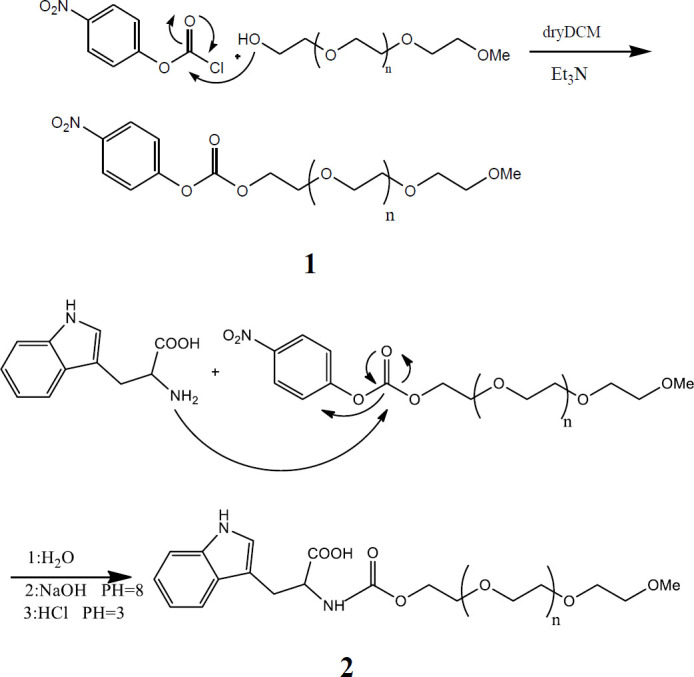
Commonly activated mPEG (2 and 5 KDa) derivatives. 1: mPEG-p-nitrophenyl carbonate (mPEG-pNPC); 2: mPEG-Tryptophan (2 and 5 KDa) used for non-covalent PEGylation of proteins

**Figure 2:**
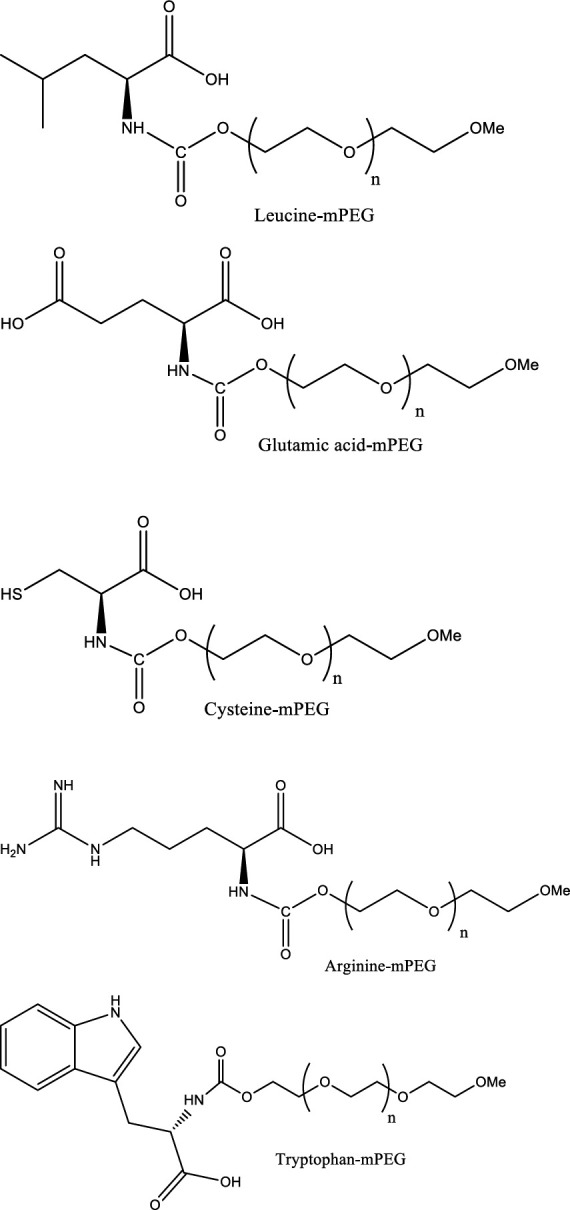
Structures of the various amino acid-mPEGs 2 and 5 KDa as excipients

The synthesized polymers (Figures 1 and 2) were analyzed by ^1^H nuclear magnetic resonance (NMR) on a Varian VXR 300-MHz spectrometer (Varian, Zug, Switzerland) after dissolution in deuterated Chloroforme (CDCl_3_). For Fourier transform infrared (FTIR) spectroscopy, pellets of 1% (w/w) of product in KBr were prepared and examined on a PerkinElmer 100 FTIR spectrometer (PerkinElmer, Schwerzenbach, Switzerland) in the range of 4000 to 400 cm-1.

***Stability studies***

Stability studies were performed based on the previous studies (16). Briefly, the samples (hGH and aa-mPEG-hGH) were dissolved in phosphate buffer (50 mM, and pH=7.4) to achieve a concentration of 0.1 mg/ml and 0.01mg/ml with different protein:m-PEG ratios (Table 1). The samples were then stored at three different temperatures, refrigerator (4 ^°^C), room temperature (25 ^°^C), and 37 ^°^C. The samples were analyzed by size exclusion chromatography (SEC) for 1, 4, and 8 weeks to evaluate the physical instabilities. The SEC analysis method was used to determine the amount of monomer, dimer, and related substances with higher molecular weights, including oligomer and polymer. A set of HEWLETT PACKARD HPLC (1100 SERIES) with UV detector and Alltech (Macrospher GPC 100 A, ID 7.5 mm, 300 mm, Biosep- SEC-S 2000, USA) column were used for SEC-HPLC analysis. The mobile phase consisted of phosphate buffer (0.063 M, pH 7.0) and 2- propanol, with percentages of 97% and 3%, respectively. The injection volume was 20 ml. The flow rate was 0.6 ml/min, and the effluent was monitored at 214 nm.

***Structural properties***

A J815 spectrometer (Jasco, Tokyo, Japan) was used for all circular dichroism (CD) measurements with a Jasco 2-syringe titrator under constant nitrogen flush at room temperature. The instrument was controlled by Jasco Spectra Manager ^TM^ software. The scanning speed was 50 nm min ^-1^, the response was set to 2 sec, and the bandwidth was set to 1 nm. All spectra were taken at 20 ^°^C in a quartz cuvette with a 1 mm path length. The samples of hGH:aa-mPEG and native hGH were prepared in 50 mM phosphate buffer at pH 7.0 and using a solution containing 0.1 mg/ml for both hGH:Glu-mPEG and hGH. Far-UV CD spectra were obtained over a wavelength range of 190-240 nm with an average of 5 scans. The data at each wavelength were averaged for 5 sec. The sample cell path length was 1 mm.

***Molecular simulation***

The structure of hGH was obtained from the RCSB protein data bank (1HUW), and the structure of polymers in molecular weight of 2 KD was built in MOE software. The structures of polymers were preliminarily minimized energy, which was saved as a PDB file. To investigate the activity of polymers, docking studies were performed in the catalytic site of the hGH (PDB ID: 1HUW) by the MOE program. After analyzing docking scores, we selected the most potent compound for further molecular dynamic (MD) studies.

***Evaluation of excipients cytotoxicity***

The Nb2-11 cell line was used in the present study to evaluate the cytotoxicity of excipients (17). The cells were prepared in RPMI 1640 medium (Biosera, East Sussex, UK) plus 10% fetal bovine serum (FBS) (Biosera), 10% horse serum (HS) (Biosera), and 1% penicillin-streptomycin (Gibco/Invitrogen) in an atmosphere of 5% CO_2_, 95% air at 37 ^°^C. Nb2-11 cells proliferated according to the concentration of hGH. Cell proliferation was analyzed using the 3-(4,5-dimethylthylthiazol-2-yl)-2,5-diphenyltetrazolium bromide (MTT) assay. All measurements were performed in triplicate. Cells were seeded in 96-well plates at 8000 cells per well and cultured. The plates were incubated overnight at 37 ^°^C. After incubation, samples were centrifuged with a refrigerated centrifuge (5 min at 1,500 rpm) at full speed. All supernatant was collected into a new centrifuge tube and prepared with fresh serum-free media in different concentrations of amino acid-mPEGs. In addition, a control group without drug treatment was also considered until comparisons between the control group and the mentioned groups. After 48 hr of incubation, the samples were centrifuged again, the cell culture medium was removed, 0.5 µl of the MTT reagent was added to individual wells, and cells were incubated for 4 hr. After centrifugation, the supernatant was removed from the wells, and 200 μl/well DMSO was added. After being placed on a shaker for 15 min and in dark conditions, the Eliza reader was used to measure absorbance at 570 and 630 nm. Cell numbers were determined using a standard curve plotted from a linear relationship between cell number and absorbance.

***Statistical analysis***

Statistical analysis was done using GraphPad Prism 8 (GraphPad Software, Inc.). All tests were performed at least in triplicate. Data are expressed as mean±SD. A three-way analysis of variance (ANOVA) followed by a Tukey *post-hoc* test was used for testing overall group differences. Differences between means were considered statistically significant if the *P*-value was less than 0.05.

## Results

***Characterization of the PEG Derivatives***

**Figure 3:**
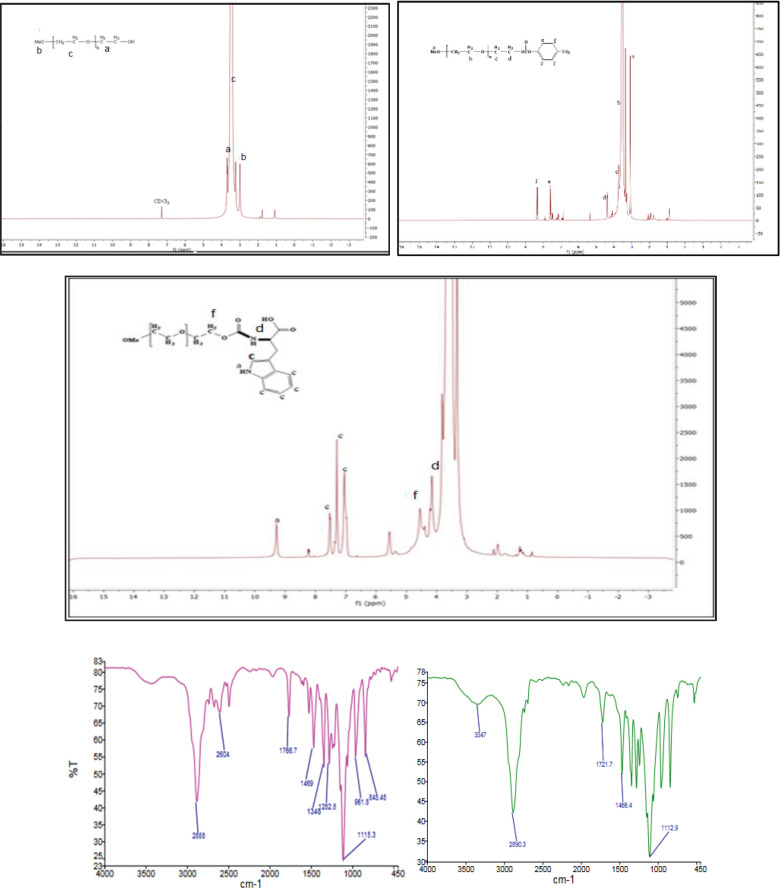
^1^H NMR analysis mPEG showed CH_2_OH near 3.8 ppm after forming a carbonyl group, and the carbamate group shifted to 4.3 and 4.5 ppm. The carbonyl peak in 1766 cm^−1^ shifted to 1721 cm^−1^, which indicates that the carbonyl groups can then be converted into carbamate groups, and amino acid-mPEGs were successfully synthesized

After the reaction of p-nitrophenyl carbonate-mPEG (4npc-mPEG) with amino acids (Trp, Glu, Leu, Arg, and Cys), the carbonyl peak in 1770 cm^−1^ shifted to 1719 cm^−1^, indicating that the amino acid-mPEGs were successfully synthesized. The resulting carbonyl groups could then be converted into carbamate, as shown in Figure 3. Also, ^1^H NMR analysis mPEG indicated the formation of carbonyl groups and then converted to carbamate group. ^1^H NMR analysis mPEG showed CH_2_OH near 3.8 ppm after forming a carbonyl group, and the carbamate group shifted to 4.3 and 4.5 ppm. At this point, identification of the proton of carbonyl and carbamate peak by FTIR in the first step was clear, and then the carbamate and carbonyl signal was confirmed by NMR.

***Stability studies***

In the present study, the effect of different amino acid-mPEGs as excipients on the physical stability of hGH was investigated and compared with hGH alone. Two different molar ratios of protein: excipient were used for these purposes. The results showed that the molecular weight of mPEG directly affects the degradation percentages of protein monomers. Five solutions were prepared: (1) hGH with no amino acids (control), (2) 50 mM amino acid-mPEGs 2 kDa, (3) 50 mM amino acid-mPEGs 5 kDa, (4) 50 mM mPEG 2 kDa, and (5) 50 mM mPEG 5 kDa. Protein samples were stored at different temperatures (4 ^°^C, 25 ^°^C, 37 ^°^C), and the stability studies were performed in intact formulations and the formulation containing Glu-, Trp-, Leu-, Arg- and Cys-mPEGs 2 and 5 kDa and mPEG alone in a 1:1 and 1:10 molar ratio over 1, 4, and 8 weeks. The results were evaluated by the SEC method every week to determine the effect of amino acid-mPEGs on the stability of proteins. hGH was dissolved in 50 mM sodium phosphate buffer (pH 7.5) at 4 ^°^C. 

(1) In the first week, it was observed that the stability increased in amino acid-mPEG 5 kDa with 1:1 and 1:10 molar ratios of protein to amino acid-mPEG compared with amino acid-mPEG 2 KDa 1:1 and 1:10 molar ratios. Also, the highest stability was achieved by adding the 1:10 molar protein ratio to Cys and Arg-mPEG 5 kDa. 

(2) In the fourth week, a 1:1 molar ratio of protein to amino acid-mPEG 2 kDa increased the stability of hGH in contrast with amino acid-mPEG 5 kDa. When excipients were added to protein and stored at 4 ^°^C), Glu first had the highest stability, then Trp and mPEG alone, followed by Leu, Arg, and Cys. It should be mentioned that Arg and Cys did not increase the stability at 4 weeks and even led to reduced stability by increasing the time.

(3) In the eight weeks, a 1:1 molar ratio of amino acid-mPEG 2 kDa led to increased physical stability compared with amino acid-mPEG 5 kDa. When adding Glu as a head group to the polymer, the highest percentage of monomer was reached after eight weeks compared with 1 to 4 weeks. However, after Glu at the same time, Trp-mPEG 2 kDa with a molar ratio of 1:1 increased the stability of hGH more than Leu, Arg, Cys, and mPEG alone.

**Figure 4:**
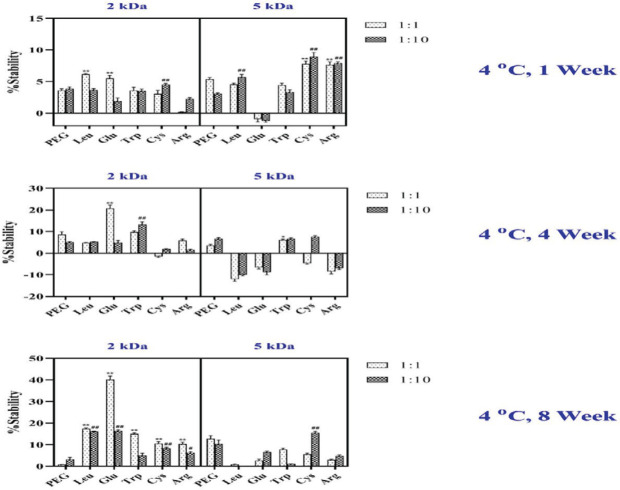
Stability was changed by the addition of Glu-, Trp-, Leu-, Arg- and Cys-mPEGs 2 and 5 kDa in addition to mPEG alone in a 1:1 and 1:10 molar ratios over 1, 4, and 8 weeks in 50 mM sodium phosphate buffer (pH 7.5) at 4 ^°^C. Data are expressed as mean±SD (n=3). *P<0.05 and ** *P<*0.001, compared with mPEG 1:1 molar ratio; ^#^ *P<*0.05 and ^## ^*P<*0.001 compared with mPEG 1:10 molar ratio

The molecular weight of mPEG polymer was shown to have an important role in increasing the physical stability of hGH. Stabilization to a higher degree was detected using mPEG 2 kDa when stored at 4 ^°^C. The best stability was obtained with Glu-mPEG 2 kDa-hGH with a molar ratio of 1:1 at 4 ^°^C (Figure 4).

**Figure 5:**
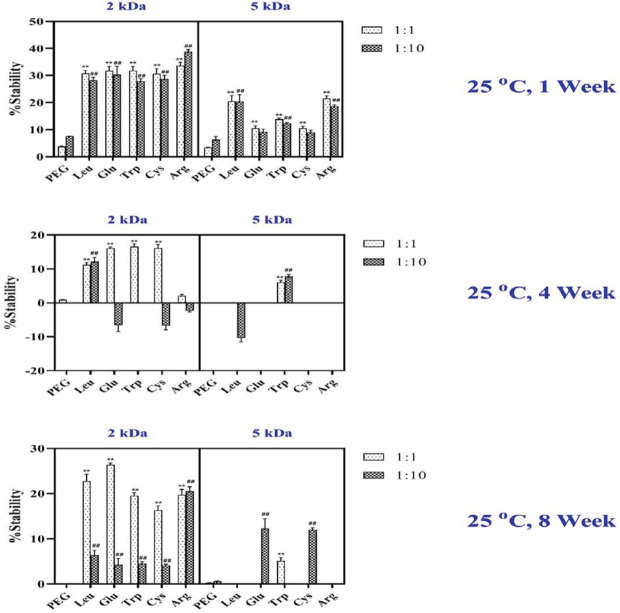
Physical stability of hGH was evaluated by measuring the changes in the percentage of monomer overtime at 25 ^°^C. Data are expressed as mean±SD (n=3). **P<0.001, compared with the mPEG 1:1 molar ratio; ^## ^*P<*0.001, compared with the mPEG 1:10 molar ratio

The physical stability of hGH was evaluated by measuring the changes in the percentage of monomer overtime at 25 ^°^C. Equimolar amounts of L-Arg, L-Trp, L-Leu, L-Glu, and L-Cys- mPEGs 2 and 5 kDa and mPEG 2 and 5 kDa all enhanced hGH stability as compared with hGH only (Figure 5). hGH stabilization, physical stability, or change in monomer percentage was observed using Glu-mPEG (2 kDa) and Trp–mPEG (2 kDa).

The formulations were stored at room temperature and contained 2 or 5 kDa PEG moieties. Consequently, the observed differences in hGH stabilization might be more likely ascribed to the head group rather than the mPEG moiety. Storage at room temperature resulted in a monomer loss for all samples during 4 and 8 weeks, except samples stored for one week (Figure 5). 

(1) In the first week, a 1:1 and 1:10 molar ratio of protein to amino acid-mPEG 2 kDa was observed compared with the 1:1 and 1:10 molar ratio of protein to amino acid-mPEG 5 kDa, which led to increased stability at room temperature. The highest increased stability was achieved by adding the 1:1 molar protein ratio to Glu, Leu, and Trp -mPEG 2 kDa. In contrast, the intensity of stability reduced over time with the addition of mPEG alone. 

(2) Following Storage for 4 weeks at 25 ^°^C for a 1:1 molar ratio of protein to amino acid-mPEG 2 kDa, the physical stability increased in contrast with amino acid-mPEG 5 kDa. When excipients were added to protein and stored at 25 ^°^C for 4 weeks, Glu demonstrated the highest stability, followed by Trp and Leu-mPEG 2 kDa. In addition, Trp-mPEG 5 kDa had positive effects on the physical stability of hGH. Arg, Cys-mPEG 2 kDa (at 1:1 molar ratio), and Glu-mPEG 2 KDa (at 1:10 molar ratio) reduced physical stability at 25 ^°^C for 4 weeks. Additionally, mPEG 2 and 5 kDa alone and Glu, Cys, and Arg-mPEG 5 KDa did not change the physical stability, and Leu-mPEG 5KDa induced unfavorable change. 

(3) In 8 weeks, for a 1:1 molar ratio of amino acid-mPEG 2 kDa, physical stability increased compared with amino acid-mPEG 5 kDa and mPEG 2 and 5 kDa alone. When adding Glu as a head group to a protein, the highest percentage of monomer was achieved, followed by Leu and Trp-mPEG 2 kDa after 8 weeks; therefore, the stability of hGH is highly dependent on head groups. The hGH alone as the control without any modification was not stable even for a week at 25 ^°^C, while the stability was prolonged up to 8 weeks by using Glu-mPEG, Trp-mPEG, and Leu-mpEG 2 KDa.

According to SEC results, the effects of different excipients on monomer degradation in all samples following storage at different times and temperatures were shown in Figures 4 and 5. 

**Figure 6:**
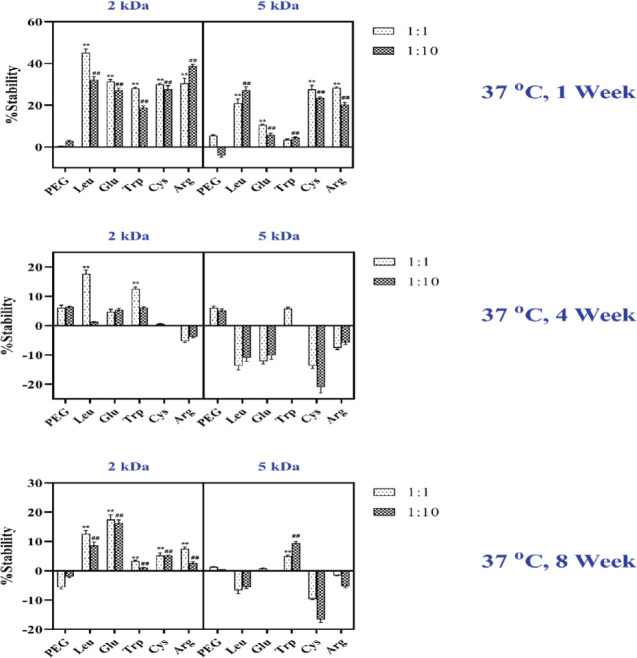
Physical stability of hGH was evaluated by measuring the changes in the percentage of monomer overtime at 37 ^°^C. Data are expressed as mean±SD (n=3). **P<0.001, compared with the mPEG 1:1 molar ratio; ^##^*P<*0.001, compared with the mPEG 1:10 molar ratio

Studies were performed in sodium phosphate buffer (pH 7.4) using 10 mg/ml hGH at 37 ^°^C by adding amino acid-mPEG 2 and 5 kDa (Figure 6). hGH was stable over a week, and all excipients significantly increased the stability, as shown by the increased monomer percentage in the chromatogram. An increase in stability of hGH to a lesser extent was obtained with mPEG alone. The hGH physical stability studies with all the excipients, including mPEG, were performed at 4 weeks of incubation at 37 ^°^C. The results of 4 weeks of incubation were comparable with the storage temperature of 25 ^°^C. In 4 weeks, a 1:1 molar ratio of protein to amino acid-mPEG 2 kDa increased the physical stability of hGH in contrast with amino acid-mPEG 5 kDa. When excipients were added to protein and stored at 25 ^°^C for 4 weeks, Leu-mPEG 2KDa with a 1:1 molar ratio had the highest stability. Then, mPEG alone and Glu followed Trp-mPEG 2KDa increased physical stability. It should be mentioned that Arg-mPEG 2KDa and Cys, Leu, Glu, and Arg-mPEG 5 KDa did not increase stability at 4 weeks and even led to reduced stability and appeared as an aggregation peak on the SEC graph.

After 8 weeks, a 1:1 molar ratio of amino acid-mPEG 2 kDa, the stability increased compared with amino acid-mPEG 5 kDa except for Trp-mPEG 5KDa and mPEG 5kDa alone. When adding Glu as a head group to the protein, the highest percentage of monomer was followed by Leu and Arg-mPEG 2 kDa after 8 weeks. Glu, as a head group with a molar ratio of 1:1, increased the stability of hGH and achieved a higher degree compared with 4 weeks (Figure 6).

***Molecular simulation***

The structure of hGH was obtained from the RCSB protein data bank (1HUW), and the polymers’ structure with a molecular weight of 2 kDa was built in MOE software. The energy of modified structures was preliminarily minimized and saved as a PDB file. To investigate the activity of polymers, a docking study was performed in the catalytic site of hGH (PDB ID: 1HUW) by the MOE program. After the result analysis of docking scores, the most potent compound was selected for further molecular dynamic (MD) studies.

MD simulation was performed to investigate the interaction of protein (1HUW) with amino acid:polymer mPEG (three amino acids, including Glu, Leu, and Trp). The stability and interaction of structure were also studied via simulation. Each MD simulation has been carried out for 90 ns and repeated three times by using NAMD 64 package and charm force field for the system model. Analyses of molecular conformation were depicted with the VMD package. Root-mean-square derivations (RMSD) is a numerical measurement and the fastest way that can be used to identify significant changes in protein structure compared with the starting point. It also allows tracking dynamic changes following protein modification. A leveling off or flatting of the RMSD curve can also indicate that the protein has equilibrated. 

**Figure 7:**
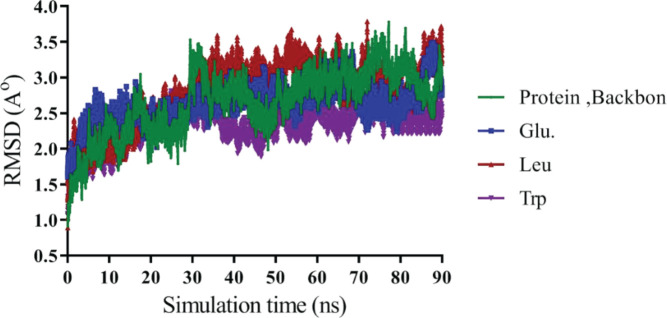
Root-Mean-Square Deviation plots of the protein backbone, Glutamic acid, Tryptophan, and Leucine

As shown in Figure 7, RMSD plots and system stability were measured and plotted for Glu, Trp, protein backbone, and Leu. After approximately 90 ns of simulation, all samples reached stability after about 30 ns of simulation. The mean RMSD value for protein backbone and protein-ligand complex that includes Glu, Trp, and Leu conjugated to mPEG 2 kDa was 2.67±0.45 Å, 2.68±0.30 Å, 2.3±0.27 Å, and 2.8±0.17 Å, one-to-one.

**Figure 8:**
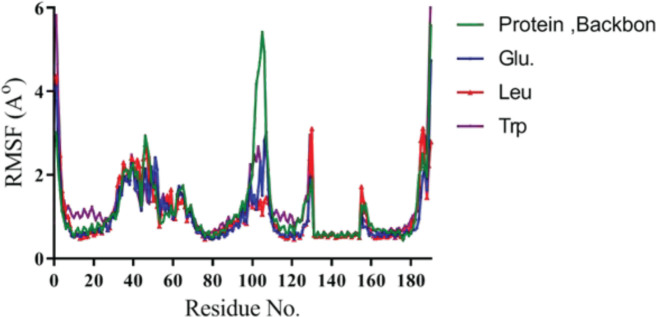
Root Mean Square Fluctuation of the protein backbone, Glutamic acid, Tryptophan, and Leucine molecules

The RMSF plots of ligand-bound protein and protein backbone are illustrated in Figure 8. The protein structures of the three excipients had similar RMSF distribution and the same trends of dynamic features. The average RMSF of the protein backbone and protein-ligand complex, including Glu, Trp, and Leu conjugated to mPEG 2 kDa was 1.29±0.93, 1.00±0.66, 1.22±0.8, and 1.04±0.66 Å, one-to-one. Lower RMSF values of ligand-bond protein were found for residues located in the binding site, and the high peak of fluctuating shows that residues are situated far from the active site. As shown in Figure 8, stabilizing excipients help protein backbone fluctuations around amino acid number 100 to render stability.

**Figure 9:**
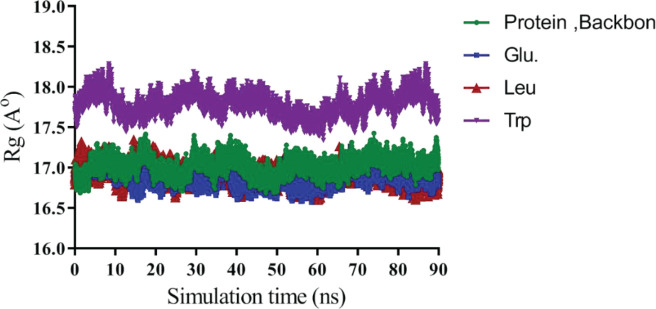
The radius of gyration of the protein backbone, Glutamic acid, Tryptophan, and Leucine molecules

**Figure 10:**
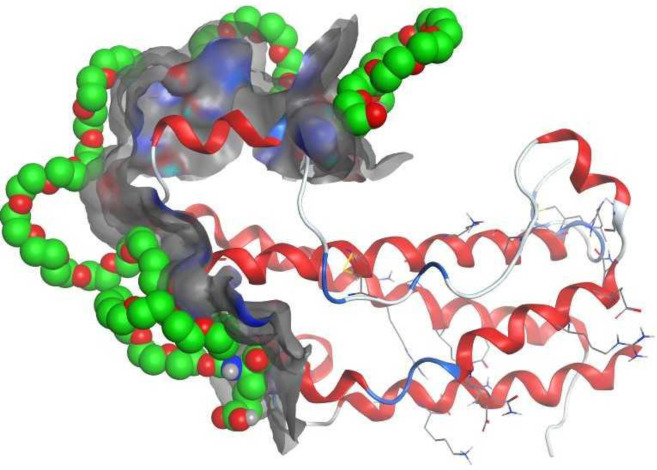
Interaction of the surface charge of growth hormone and Glu-mPEG 2KDa as noncovalent PEGylation

The radius of gyration (Rg) is a parameter that shows the compactness of protein structure and describes the bound and non-bound systems equilibrium conformation. As shown in Figure 9, Rg values of the protein backbone and Glu-mPEG were17.02±0.11 and 16.88±0.1A °, so Leu-mPEG and Trp-mPEG, which were 16.92±0.1A ° and 18.48±1.97 A ° respectively. Figure 10 indicates the surface charge of hGH was positive and interacted with Glu-mPEG 2KDa as noncovalent interaction.

***Evaluation of the structural properties***

**Figure 11:**
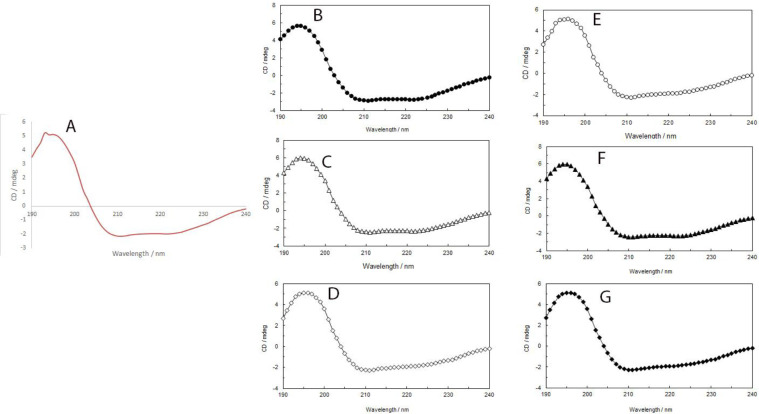
hGH secondary structure at different storage conditions. (A) At 4 ^°^C and zero time, (B) At 4 ^°^C, t=4 wk, (C) At 25 ^°^C, t=4 wk, (D) At 37 ^°^C, t=4 wk, (E), At 4 ^°^C, t=8 wk, (F) At 25 ^°^C, t=8 wk, (G) At 37 ^°^C, t=8 wk

The structural properties of Glu-mPEG: hGH were analyzed by the CD method and compared with native protein stored in the same conditions. Glu-mPEG: hGH and hGH were prepared in 50 mM phosphate buffer at pH 7.4 and using a solution containing 0.1 mg/ml, and far-UV CD spectra were obtained over a wavelength range of 190-240 nm with an average of 5 scans. Glu-mPEG 2KDa and hGH were stored at 4 ^°^C, 25 ^°^C, and 37 ^°^C for 4 and 8 weeks to evaluate the secondary structure. The results (Figure 11 And Table 2) show the CD spectra and distribution of the secondary structure elements of Glu-mPEG 2KDa in reaction media. These data indicated that conformational properties of Glu-mPEG 2 KDa with a 1:1 molar ratio had altered at 25 ^°^C and 37 ^°^C in 4 and 8 weeks.

***In vitro biological activity***

**Figure 12:**
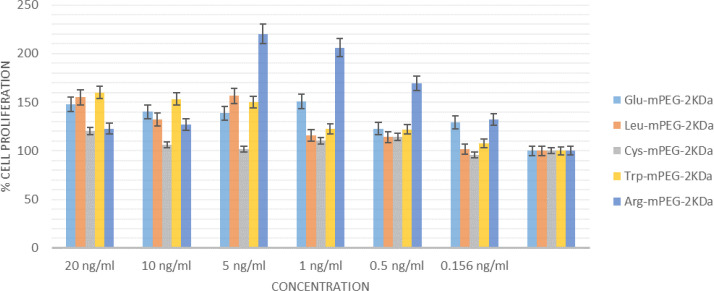
Nb2 bioassay. After 48 hr of incubation of various concentrations of the five different amino acid-mPEGss 2 KDa

In vitro biological studies were carried out using NB2-11 cells by MTT assay. This method stimulated the NB2-11 cell growth and replication by hGH. Most of the target compounds showed significant proliferative activity with no cytotoxic properties (Figure 12).

**Table 1:** Samples were used to analyze the physical stability of the protein (hGH). The Molar ratio and the type of aa-mPEGs were different in each ample

Sample	Molar ratio	Concentration
hGH:aa-PEG 2kDa	1:1 1:10	0.1mg/ml hGH :0.01mg/ml aa-PEG 2kDa 0.1mg/ml hGH :0.1mg/ml aa-PEG 2kDa
hGH:m-PEG 2kDa	1:1 1:10	0.1mg/ml hGH :0.01mg/ml m-PEG 2kDa 0.1mg/ml hGH :0.1mg/ml m-PEG 2kDa
hGH:aa-PEG 5kDa	1:1 1:10	0.1mg/ml hGH :0.025mg/ml aa-PEG 5kDa 0.1mg/ml hGH :0.25mg/ml aa-PEG 5kDa
hGH:m-PEG 5kDa	1:1 1:10	0.1mg/ml hGH :0.025mg/ml m-PEG 5kDa 0.1mg/ml hGH :0.25mg/ml m-PEG 5kDa

Root-mean-square fluctuation (RMSF) is a numerical measurement similar to RMSD. Still, instead of the positional difference between the entire structures over time, it indicates the macromolecular flexibility, local changes in the protein structure, and dynamic measurements for every atom or residue

**Table 2:** hGH secondary structure elements at different conditions

System	alpha-helix%	Beta-sheet%	Turn%	Unordered Coil%
hGH (4 °C, zero time)	51.05 ± 0.3	21.47 ± 0.3	1.75 ± 0.3	25.73 ± 0.3
hGH (4 °C, 4 weeks)	49.23 ± 0.46	19.42 ± 0.46	2.75 ± 0.46	28.60 ± 0.46
hGH (4 °C, 8 weeks)	49.16 ± 0.48	19.37 ± 0.48	2.74 ± 0.46	28.73 ± 0.46
hGH (25 °C, 4 weeks)	48.05 ± 0.47	19.27 ± 0.47	2.75 ± 0.47	29.93 ± 0.47
hGH (25 °C, 8 weeks)	47.96 ± 0.45	19.31 ± 0.45	2.77 ± 0.45	29.96 ± 0.45
hGH (37 °C, 4 weeks)	47.91 ± 0.47	19.31 ± 0.47	2.77 ± 0.47	30.01 ± 0.47
hGH (37 °C, 8 weeks)	47.71 ± 0.51	19.33 ± 0.51	2.75 ± 0.51	30.21 ± 0.51

## Discussion

The instability of therapeutic proteins in solution may lead to protein denaturation or aggregation and affect the therapeutic products’ efficacy and safety (18). Also, protein aggregation resulting from undesirable protein-protein interaction can lead to the loss of therapeutic effects and may induce harmful immune responses (19, 20). Numerous external factors, including storage temperatures, solution buffers, and pH, can negatively impact the conformational and physical stabilities of proteins (21). Therefore, the correct selection of buffer, pH, and excipient is essential to improve protein stability. The buffer that is used to modulate the pH of a solution is a determinant factor in proteins’ structure, stability, and biological activity (22). Excipients are ingredients added to therapeutic products to improve bioavailability and protein-based pharmaceuticals’ stability. Evidence suggests that some free amino acids can be used as excipients to improve protein solubility and inhibit aggregation (23, 24).

The present study aimed to enhance the physical stability of the hGH protein by adding novel synthesized excipients. Various amino acids were conjugated with mPEG at different molecular weights (2 and 5 kDa), and the physicochemical properties and stability profile of hGH were investigated. So the chemical reactions were performed in two steps, as shown in Figure 1. Activated mPEG is a necessary reagent that has been widely used for the PEGylation of protein. A single-step method is described to activate PEG for binding to amino acids, where phenyl chloroformate is an activating reagent. It was a one-step procedure with high purification and was prepared at room temperature. Its relative stability in a neutral aqueous solution is reactive enough to react quickly with amino acids in the aqueous phase.

After the reaction of the intermediate of 4npc–mPEG with amino acids and confirming new excipients, the carbonyl group is a perfect functional group for detection by FTIR spectroscopy. As seen in Figure 3, the carbonyl peak in 1770 cm^−1^ shifted to 1719 cm^−1^, indicating that the amino acid-mPEGs were successfully synthesized. Here, proton NMR studies were used to confirm carbamate formation by the carbonyl group’s intermediation. (25, 26). ^1^H NMR spectroscopy analysis showed the CH_2_OH peak was near 3.8 ppm, and after forming a carbonyl group, the carbamate group shifted to 4.3 and 4.5 ppm.

In the present study, five different amino acids, including Trp, Arg, Cys, Glu, and Leu, were used for conjugation with mPEG to improve the stability of hGH formulations. Arg and Glu have been combined to form synergistic protein-stabilizing effects on various protein solutions (27). It should also be noted that Arg can increase protein solubility and stability and dramatically reduce the viscosity of highly concentrated protein solutions (14). In contrast, Cys is widely used as the most reactive residue because the thiol group is a strong nucleophilic head group (28). Pharmaceutical polymers have been widely used to improve the stability and bioavailability of pharmaceutical products and control drug release. The most common carrier for polymer therapeutic is PEG (7). 

Protein stabilization was studied each week by the SEC method. hGH was stable in 50 mM sodium phosphate buffer (pH 7.4) at 4 ^°^C. The formulation was changed by adding Glu, Trp, Leu, Arg, and Cys-mPEGs 2 and 5 kDa and mPEG alone in 1:1 and 1:10 molar ratios, and the stability profile was assessed during 8 weeks at certain intervals. 

Storage of hGH in the presence of aa-mPEGs at 4 ^°^C for 1, 4, and 8 weeks did not change the stability of hGH remarkably compared with hGH alone as the control group. Muller *et al*. studied the influence of the molecular weight of the mPEG moiety on protein stability. They indicated that increasing the molecular weight of the mPEG sterical hindrance plays a central role in the stability and leads to reduced interactions between the respective head group and protein (15).

In another study, the effect of physical PEGylation on insulin’s structural and colloidal stability has been investigated. The results indicated that physical PEGylation led to beneficial effects on the stabilization and shielding of the insulin structure, which is not prone to fibrillation and aggregation (29).

Monomer percentage of hGH at different aa-mPEG 2 kDa molar ratios revealed that the increase of stability by increasing the storage time was observed even at a 10:1 molar ratio of aa-mPEG 2kDa to protein at 4 ^°^C (Figure 4). Experimental analysis revealed that one-week of mPEG 5KDa storage caused increased stability. Still, when enhancing the storage time, mPEG 2KDa stability was improved compared with mPEG 5KDa and increased monomer percentage (Figure 4). It has been previously shown by Muller *et al*. that Trp-mPEG 2 kDa effectively reduced the aggregation of protein under stress conditions, whereas Trp-mPEG 5 kDa was less effective. PEGs can remove solvate water from biopharmaceuticals used as precipitative agents (14). By increasing storage time, the molecular weight of PEG may lead to high precipitation and reduce protein stability. (14)

The most stable formula was observed when Glu-mPEG 2 kDa at 1:1 was added to the hGH formulation (Figures 4 to 6). 10-fold increase in the stability of hGH was detected only by adding Glu-mPEG 2 kDa as compared with mPEG 2 kDa alone, indicating that the molar ratio of head groups was a determinant factor in the stability of the protein. Furthermore, using 1:1 compared with 1:10 mass ratio of other excipients may have inhibited the monomer degradation significantly. This stability increased by adding different new excipients in a 1:1 molar ratio over eight weeks. 

Non-covalent PEGylation of insulin with a PEG derivative, CB[7]-PEG, could stabilize the protein in the formulation. It was shown that aggregation of insulin was reduced following agitation when CB[7]-PEG was added to the formulation and was stable over the next 100 days (30). 

Reduced stability would be observed for mPEG5 kDa. The strongly decreased stability at room temperature belongs to mPEG: protein 5 kDa at 1:10 molar ratio (Figure 5). It was also reported that an increase in the content of PEG side groups provided greater stability of avidin-PPEG complexes at high protein loadings (31). This observation was in line with the present study and indicated the lower molecular weight of the polymer was more effective in reducing the aggregation.

In this article, the interactions of the Arg with hGH on protein stabilization and interaction through its guanidinium group were studied. In the previous study, Arg was used to suppress protein aggregation, but it can enhance aggregation depending on protein concentration and type (32). In the present study, Arg-mPEG was added at an equimolar ratio or 10-fold Arg-mPEG: protein. The results showed that Arg could not increase protein stability as well as Glu, Trp, or Leu-mPEG. Glu can potentially increase the physical stability of protein formulated at neutral pH under accelerated stability conditions. 

The secondary structure of protein plays an important role in protein structure and folding, so CD studies were applied to ensure the secondary structures of protein remain intact. The hGH secondary structure in the presence of Glu-mPEG 2 KDa and the native hGH were evaluated when stored at 4 ^°^C, 25 ^°^C, and 37 ^°^C. The CD spectra of Glu-mPEG: hGH and hGH showed that the PEGylation processes were not destructive, and the secondary structure of the protein was preserved. CD study suggested structural conformation of the protein model was not altered following non-covalent PEGylation (33). The results of another study indicated that less change in the CD measurements of the non-covalent PEGylated catalase as compared with those of covalent PEGylation was observed, suggesting less influence on protein conformation (34).

The Nb2 bioassay measures the somatotrophic activity of hGH. Their activities were examined to study the potential application of the amino acid-mPEG 2 kDa as potential excipients for protein formulations. After 48 hr of incubation, various concentrations of the five different amino acid-mPEGs 2 KDa, including Glu, Trp, Arg, Cys, and Leu, showed significant proliferative activity (Figure 11). From the point of view of structure-activity relationship, all of the synthesized compounds in this study had similar effects on stimulating cell proliferation. It should be noted that upon covalent PEGylation of hGH, the resultant molecule showed antagonistic activity in cell-based bioassay (35). Other researchers previously reported preserving the biological activity of the protein and peptide model following non-covalent PEGylation (30, 34, 36, 37). Additionally, the enzyme activity of α-amylase in PEG-b-PAMA as a non-covalent agent was not inhibited (38).

In molecular dynamics study, stability of the secondary structures and conformational changes in protein alone (backbone) over time as compared with protein-ligand complex. All RMSD were above 2 Å. Also, Figure 7 shows that RMSD for Leu was much higher than Trp and Glu, which suggested the opening of 1HUW protein and dispersing of protein. It seemed reasonable that Glu and protein systems had similar RMSD distribution and the same trends of dynamic features. Also, previous studies by Muller *et al*. have shown that Trp excipient increases protein stability, which agrees with present results. The RMSF values for residues located in the binding site, which explained by the relatively stronger binding of a ligand with these residues that create a change in protein flexibility around amino acid number 100. The RMSF analysis showed excipients restore protein stability by being situated in the active site. Higher Rg values confirmed the opening of hGH chains in the presence of these excipients, resulting in the release of Trp-mPEG from the protein. It should be noted that this difference was not as great as others and could be related to the aromatic rings of Trp-mPEG, which are larger than leu-mPEG and Glu-mPEG.

Figure 10 indicates the surface charge of hGH, and most of the positive charge is found near the surface. Glu-mPEG with a negative charge could increase protein stability. The MD studies can well explain our experimental results and corroborate this notion that the function of Glu-mPEG is an excipient that acts better than others.

## Conclusion

It was founded that mPEG did not act as a suitable excipient, so new head groups are needed to suppress monomer loss. Therefore, Glu, Trp, Leu, Arg, and Cys could slow the kinetics of monomer degradation without the need for covalent conjugation of PEG. Significant increasing stability was observed with Leu-mPEG, Trp-mPEG, and Glu-mPEG 2 kDa with a 1:1 molar ratio. The type of head group in mPEG polymers, the polymer’s molecular weight, and the molar ratio of polymer to protein directly affected the protein’s stability profile. hGH as a control group is known to be stable at 4 ^°^C; a pronounced change in monomer degradation is observed when stored at 25 ^°^C and 37 ^°^C. In this study, measurements of the percentage of monomer by SEC over time demonstrated that adding a 50 mM hGH: Glu-mPEG 2 kDa with the molar ratio of 1:1 to the protein solution can significantly increase the physical stability of protein and reduce aggregation peak over storage time. The dependency of concentration (2 kDa and 5 kDa) shows the molecular weight of the mPEG polymer plays an important role in protein stability. In our study, Leu, Glu, Arg, Cys, and Trp-mPEG 2 kDa were superior to mPEG in stabilizing hGH against losing monomer. Significant stability was observed with Glu-mPEG, Trp-mPEG, and Leu-mPEG 2 kDa with a 1:1 molar ratio. 

## Authors’ Contributions

BK and MRS designed the experiments; SV performed experiments and collected data; JC, ZAT, HH, FM, and SMK discussed the results and strategy; MRS supervised, directed, and managed the study; SV, JC, ZAT, HH, FM, SMK, BK, and MRS approved the final version to be published.

## Conflicts of Interest

The authors have no interests to declare.
